# A Challenging Case of Weil's Syndrome in New York City: A Short Review of the Renal Pathophysiology and Diagnosis of Leptospirosis

**DOI:** 10.7759/cureus.88552

**Published:** 2025-07-22

**Authors:** Yone M Lwin, Thin Thin Soe, Muhammad Azhar, Isha Puri, Sonalika Agarwal, Mary Mallappallil

**Affiliations:** 1 Nephrology, State University of New York Downstate Medical Center, Brooklyn, USA; 2 Nephrology, New York City Health and Hospitals/Kings County Hospital Center, Brooklyn, USA; 3 Internal Medicine/Nephrology, State University of New York Downstate Medical Center, Brooklyn, USA

**Keywords:** ards, bile cast nephropathy, continuous renal replacement therapy, cytokine storms, hemodialysis, kidney failure, leptospirosis, leptospirosis with severe clinical manifestation, respiratory failure, rhabdomyolysis

## Abstract

Leptospirosis is a zoonotic spirochetal infection endemic to warm climate regions, with increased incidence following rainfall and flooding when sanitation is compromised. In comparison, infection rates in the United States are significantly lower. The bacterium is transmitted through the urine of infected animal reservoirs, most commonly rodents, dogs, horses, and pigs, through mucosal membranes or skin abrasions. Leptospirosis can affect multiple organ systems, with severity ranging from asymptomatic cases to life-threatening multiorgan failure. Patients may exhibit a biphasic course, initially experiencing mild illness followed by a brief recovery and then progressing to more severe symptoms such as jaundice, meningitis, renal failure, or pulmonary hemorrhage. Weil’s disease is the severe form of leptospirosis, characterized by hepatic dysfunction and renal failure, and is associated with high mortality. We present a case of Weil’s disease in a lifelong New York resident who required renal replacement therapy, including both continuous veno-venous hemodialysis (CVVHD) and intermittent hemodialysis (iHD), and ultimately made a full recovery.

## Introduction

Leptospirosis is a zoonotic infection, most prevalent in tropical, subtropical, and temperate regions, with the highest incidence of infections in South and Southeast Asia, Oceania, the Caribbean, and parts of sub-Saharan Africa. The worldwide estimated cases from a systematic review showed more than one million human cases worldwide annually, with almost 60,000 deaths. In the USA, the infection rate is approximately 100-150 cases annually, with the most reported cases in Puerto Rico and Hawaii [1,2], but it can become epidemic after rainfall and flooding [3]. Weil’s disease or icteric leptospirosis, a rapidly progressive multisystem failure, occurs in about 5%-10% of leptospirosis cases, with a 5%-15% mortality rate [4]. As of April 2024, there have been six reported cases of leptospirosis in New York, as per data from the New York City (NYC) government [5]. Here, we present a case of Weil’s disease requiring continuous renal replacement therapy and intermittent hemodialysis.

## Case presentation

A 59-year-old man, born and raised in New York with a history of hypertension, presented to the emergency department with generalized body aches mainly in the bilateral calves and weakness for two days. It was associated with non-bloody non-bilious vomiting, three episodes of large watery diarrhea, and subjective fever. Upon presentation, he was afebrile, and his vital signs were stable. Laboratory values were significant for thrombocytopenia, elevated creatinine, hypokalemia, and rhabdomyolysis (see Table 1).

**Table 1:** Laboratory data ICU: intensive care unit; CRRT: continuous renal replacement therapy; WBC: white blood cell; Hb: hemoglobin; Plt: platelet count; Cr: creatinine; BUN: blood urea nitrogen; CO₂: bicarbonate; CKD-EPI: Chronic Kidney Disease Epidemiology Collaboration; CPK: creatine phosphokinase; AST: aspartate aminotransferase; ALT: alanine aminotransferase; ALP: alkaline phosphatase; eGFR: estimated glomerular filtration rate

Blood test	Normal range	Admission day	Day 4 (ICU transfer)	Day 8 (CRRT initiation)
WBC	4.5-10.9 K/µL	7.56	14.12	16.49
Hb	14-18 g/dL	13.6	10.2	9.5
Plt	130-400 K/µL	111	70	139
Cr	0.7-1.2 mg/dL	2.68	3.82	8.79
BUN	6-20 mg/dL	34	55	139
Potassium	3.5-5 mmol/L	3.1	3	4.4
CO_2_	24-31 mmol/L	26	23	18
eGFR (CKD-EPI 2021)	>60 mL/min/1.73 m^2^	26.6	17.4	6.4
CPK	20-200 U/L	2,385	10,881	647
AST	10-50 U/L	66	369	100
ALT	0-41 U/L	32	186	133
ALP	35-145 U/L	64	82	110
Total bilirubin	0.0-1.2 mg/dL	1.1	16.5	25.2

His urine sodium was less than 20 mmol/L, his urine potassium was 47.95 mmol/L, and fractional excretion of sodium was less than 1%, which correlates with pre-renal acute kidney injury (AKI). Urine analysis was bland without any casts. Respiratory panels were negative for COVID/influenza. Stool polymerase chain reaction (PCR) was positive for enteroaggregative *Escherichia coli* (*E. coli*) and enteropathogenic *E. coli*. He was initially treated symptomatically for acute gastroenteritis with AKI stage 3 vs. AKI on chronic kidney disease (CKD) for three days. Despite receiving maintenance intravenous (IV) fluids, he developed a fever, and his renal function continued to worsen, accompanied by an increase in creatine phosphokinase (CPK) to 10,881 U/L.

Given his occupation as a sanitation worker and reports of similar symptoms among coworkers, a broader differential diagnosis was considered, including leptospirosis, tick-borne rickettsial infections, acute HIV, West Nile virus, Babesia, and Lyme disease. He was empirically treated with 2 g of IV ceftriaxone and 100 mg of IV doxycycline twice daily. Extensive testing, including blood cultures, Babesia, Lyme, hepatitis B and C, HIV, syphilis, and other rheumatologic autoimmune diseases, was all negative. Initial antibody testing for *Leptospira* was negative on day 3 of hospitalization. Kidney ultrasound showed normal-sized kidneys without structural abnormalities.

On day 4 of his hospital stay, he became hypotensive with worsening of both renal and hepatic functions. He also became hypoxic to SPO_2_ of 86% requiring non-invasive ventilation (bilevel positive airway pressure (BiPAP)) and eventually had to be transferred to the intensive care unit (ICU) for management of septic shock secondary to possible leptospirosis despite an initial negative result, multiorgan failure, and acute respiratory failure with acute respiratory distress syndrome (ARDS), requiring intubation and vasopressor support. While in the ICU, IgM dot blot for *Leptospira* using enzyme-linked immunosorbent assay (ELISA) on day 8 tested positive eventually along with *Leptospira* DNA; qualitative PCR from urine from day 5 confirmed his diagnosis of leptospirosis. *Leptospira* DNA PCR from blood sent three days later, however, came back negative.

Concerns for other infectious processes were raised, and bronchoscopy was done, which showed serosanguinous fluid, which was negative for pathologic cytology, microbes, or pulmonary hemorrhage due to red blood cells (RBCs) of 6,000 cells/µL. The patient experienced a drop in hemoglobin to 6.7 g/dL, requiring the transfusion of two units of packed RBCs. Concurrent thrombocytopenia raised concern for possible hemolytic uremic syndrome or thrombotic thrombocytopenic purpura secondary to *E. coli* infection or leptospirosis. However, peripheral smears showed 0-2 schistocytes and a low plasmic score of 4, which ruled out both. His thrombocytopenia and hemoglobin levels eventually improved, with both abnormalities attributed to his acute illness.

**Figure 1:**
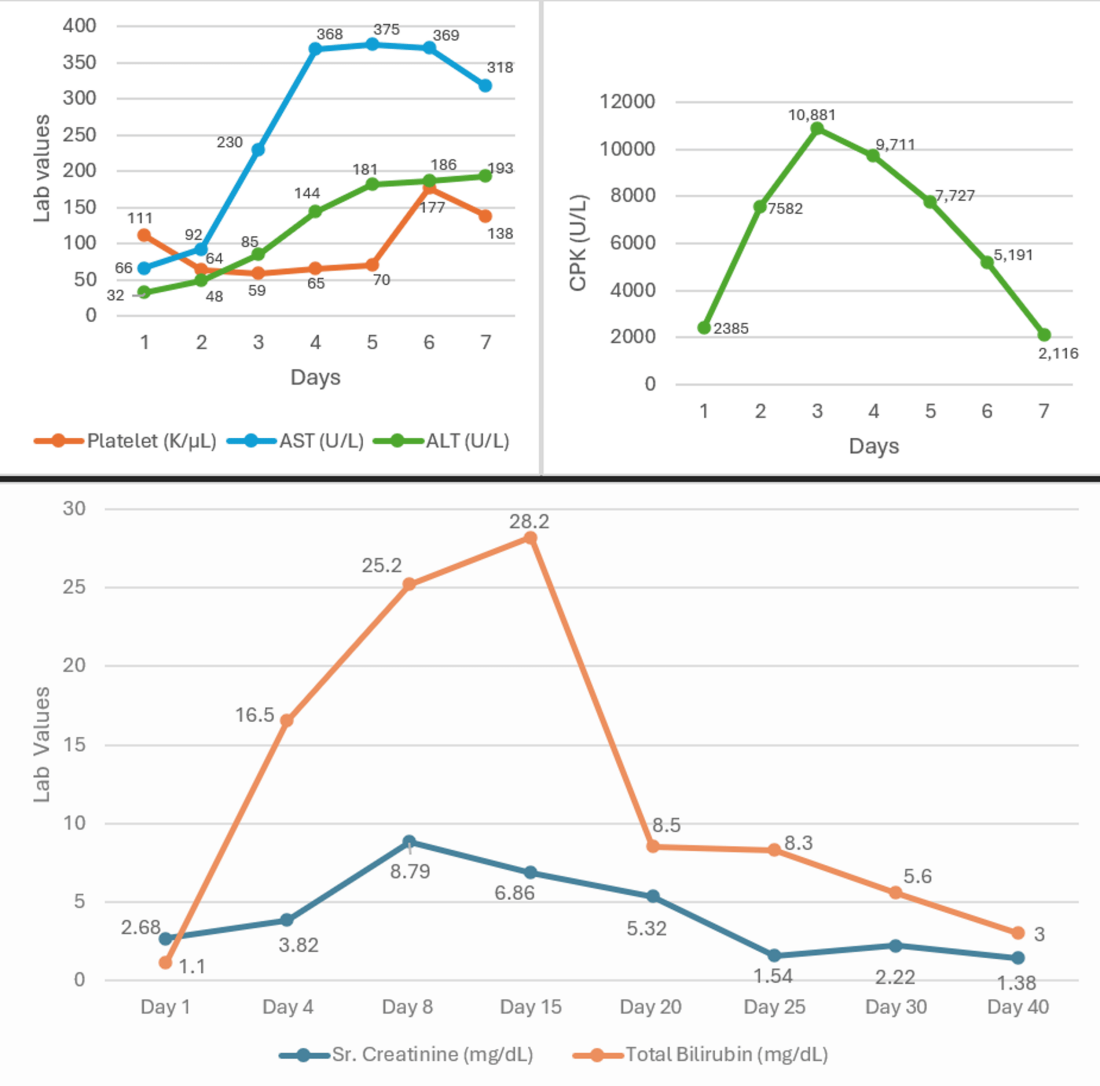
Trends of the patient’s pertinent labs throughout his hospital stays AST: aspartate aminotransferase; ALT: alanine aminotransferase; CPK: creatine phosphokinase

Even though his rhabdomyolysis resolved, his renal function continued to decline, with a creatinine level of 8.79 mg/dL, blood urea nitrogen (BUN) of 139 mg/dL, and progression to oliguria, likely due to acute tubular necrosis (ATN) from rhabdomyolysis and bile cast nephropathy secondary to hyperbilirubinemia of 29 mg/dL (see Figure 1). However, repeat urinalysis did not show any granular or bile casts.

Continuous veno-venous hemodialysis was then initiated on him for 24 hours on day 8 of hospital stay and then shifted to daily consecutive hemodialysis with 1-2 L ultrafiltration for a week. His clinical conditions, including liver function and urine output, improved steadily. Vasopressors were weaned off after two days, and he was extubated on day 5 to non-invasive ventilation, followed by transition to nasal cannula. He completed a 10-day course of ceftriaxone and doxycycline.

He had a prolonged hospital stay of 40 days with intermittent hemodialysis three times a week. As his renal function began to recover, he was monitored without hemodialysis for four days and was discharged with adequate urine output. The tunneled hemodialysis catheter was removed prior to his discharge as well. He had not required dialysis since the discharge to the subacute rehabilitation facility.

## Discussion

Clinical manifestations

Leptospirosis is caused by *Leptospira interrogans*, gram-negative, motile, coiled spirochetes with two periplasmic flagella. The motility and adhesiveness of bacteria are associated with the pathogenicity of leptospirosis; the higher pathogenic strain showed higher adhesion to kidney-cell sheets [6]. *Leptospira* colonize renal proximal tubules; hence, infected animal reservoirs such as rodents, dogs, sheep, and horses excrete bacteria through urine throughout their lifespan. It is transmitted by direct contact or indirect exposure to the urine or feces of such host animals. Symptoms usually appear 2-4 weeks after exposure. Clinical manifestations of leptospirosis are typically classified as either anicteric or icteric (Weil’s disease). Approximately 90% of cases present as the anicteric form, characterized by fever, chills, headache, myalgia, diarrhea, and vomiting. In some cases, the disease progresses to the icteric form, which may include abdominal pain, hepatomegaly, jaundice, AKI, and hemorrhagic complications. Severe cases can lead to a cytokine storm, multiorgan failure, and death. Leptospirosis has biphasic clinical presentations: acute phase and immune phase. The acute phase constitutes non-specific symptoms of the septicemic phase, fever, chills, headache, myalgia, etc., which last about 7-10 days. The immune phase is caused by antibodies to *Leptospira* such as aseptic meningitis, uveitis, or other severe forms of leptospirosis.

Pathophysiology of AKI in leptospirosis

Renal complications of leptospirosis are rare in developed countries; however, it is a known cause of AKI in developing countries, as high as 60% of the cases [7]. Renal involvement varies from mild proteinuria to severe anuric AKI requiring hemodialysis. Once a human host is exposed to pathogenic *Leptospira* through skin abrasions or oral mucosa, it disseminates in the renal proximal tubules and/or hepatocytes of humans. In our case, the patient presented to the hospital during the acute phase, and while receiving treatment, he progressed into the immune phase and developed one of the fatal complications, which is Weil’s disease, constituting ARDS and AKI.

Pre-renal components of AKI in leptospirosis are triggered by dehydration (from diarrhea, vomiting, or high fever) or hypotension caused by cytokines released from sepsis. Our patient initially had pre-renal AKI, likely from his poor oral intake and diarrhea, but later developed into intrinsic AKI, likely from multifactorial etiologies. He also had persistent hypokalemia without oliguria, which is a characteristic finding of AKI in leptospirosis. Spot urine potassium in our patient showed a moderate amount of excretion of potassium with 47.5 mmol/L. His urine potassium-creatinine ratio of 14.8 mEq/L indicated renal excretion of potassium (greater than 13). These findings are explained by increased potassium excretion through increased delivery of sodium and water in distal nephrons due to its defect in sodium and water reabsorption by proximal tubular injury [8]. Reduction in expression of NHE3 (sodium-hydrogen exchange isoform 3), increase in expression of Na+K+2CL cotransporter, and decrease in expression of aquaporin 2 play a role in these electrolyte alterations [7].

When coupled with sepsis or disseminated intravascular coagulation, it can cause oliguric or anuric AKI with hyperkalemia from ATN. Leptospirosis AKIs are often characterized by acute tubulointerstitial nephritis or ATN. Severe forms of leptospirosis are caused by dysregulated inflammation from cytokine storm followed by immunoparalysis. TNF-α, proinflammatory IL-6, chemokine IL-8, and anti-inflammatory IL-10 were known to play important roles in the pathogenesis of leptospirosis [9]. ATN is also caused by rhabdomyolysis and severe hyperbilirubinemia, which are seen in Weil’s disease. Both high levels of bilirubin and myoglobin cause direct damage to tubular epithelial cells and obstruction in kidney tubules, which exacerbates kidney injury [7]. In our patient, while serial urinalyses did not reveal granular or bile casts, it was evident that our patient suffered from cytokine storm, causing hemodynamic alterations: shock along with ATN from both rhabdomyolysis and hyperbilirubinemia.

Another feature often associated with leptospirosis is the enlargement of the kidneys seen in ultrasound with normal parenchymal echogenicity (tubulointerstitial nephritis) [7]. However, in our patient, both kidneys were <12 cm in length, which is atypical and may reflect chronic or pre-existing renal changes.

Diagnosis

Diagnosis of leptospirosis is challenging, often requiring empirical treatment with antibiotics based on clinical features before the serology results. There are multiple modalities of diagnostic testing for leptospirosis available currently, including direct methods such as microscopy (phase contrast or darkfield), histochemical staining, and immunostaining; culture techniques; molecular methods like PCR; and serologic tests such as the microscopic agglutination test (MAT), ELISA, and indirect hemagglutination assay [10].

It is possible that *Leptospira* can be isolated in blood culture if samples were obtained during the acute phase of manifestation between seven and 10 days. However, the sensitivity is low, and both false positives and false negatives are common. For MAT, live antigens of different serogroups are reacted with serum samples, and agglutination is examined by darkfield microscopy. It was regarded as the gold standard of all diagnostic techniques and was used worldwide for years with a sensitivity of 41% during the first week, 82% during the second to fourth week, and 96% beyond the fourth week of illness [11]. MAT can be positive during the immune phase of infection, which is after 8-10 days of initial presentation. CDC defined it as probable infection if the antibody titer is >200 along with clinical presentations; however, the cut-off point of titer depends on the seroprevalence of each location, with a lower titer in less common areas. Diagnostic laboratories are often required to have all the circulating types of *Leptospira* serovars, which may be costly and complex [12].

At our hospital, *Leptospira* IgM was detected using a dot blot ELISA, which targets antibodies against a broadly reactive genus-specific antigen. Antibodies on day 3 of hospital stay were negative, and antibody testing on day 8 was positive for IgM. Detection of IgM antibody by ELISA has shown about 90% sensitivity and specificity; however, it is often negative in the early phase and becomes positive from days 6 to 8, which explains our case’s initial negative test [11]. Rapid screening tests such as particle agglutination, immunodot, dipstick/comb, immunofiltration, flow-through, or lateral flow assays are readily available nowadays as well, though they offer low sensitivity.

Another widely used method is PCR assays, which detect DNA in blood or urine in the first 5-10 days after the onset of the disease and up to the 15th day. Unfortunately, for our patient, only urine qualitative PCR from day 5 came back positive, whereas blood qualitative PCR was negative at day 8 of hospital stay. Despite a negative serum PCR, the diagnosis of leptospirosis was confirmed based on clinical presentation, a positive *Leptospira* IgM by dot blot ELISA, and a positive urine PCR in our case.

Treatment

Most patients recover after the acute phase with mild symptoms that require either no treatment or only symptomatic management. Oral doxycycline, azithromycin, amoxicillin, and ampicillin have been used for outpatient treatment of mild disease, with doxycycline also serving as a prophylactic agent. IV penicillin (1.5 g every six hours), ceftriaxone (1 g once daily), and cefotaxime (1 g every six hours) have all been found to be equally effective for the treatment of severe leptospirosis when administered for a duration of seven days. Our patient was treated with both IV ceftriaxone and doxycycline to have broader coverage for 10 days, as he had a complicated hospital stay. Based on data from 2007, early initiation of daily dialysis has been shown to improve mortality significantly compared to late dialysis every other day [13]. However, a retrospective data analysis carried out in Reunion Island over 11 years did not reveal any significant improvement over the composite mortality-CKD endpoint between early and late dialysis (after 48 hours) [14]. In our patient's case, hemodialysis was initiated on the eighth day of admission, which was the 10th day since clinical presentation; we were able to successfully wean him off it.

It has also been reported that prolonged exposure or asymptomatic leptospirosis infections are associated with CKD through chronic tubulointerstitial nephritis and fibrosis without any previous history of leptospirosis-induced AKI. Severe AKI in leptospirosis is also known to increase the risk of progression to CKD during the repair and recovery process of AKI. A maladaptive repair process promotes cell inflammation followed by fibrosis of renal tubular epithelial cells through cytokines released by injured cells called IL-34, causing CKD [15].

Preventive measures for high-risk occupations

Human leptospirosis is strongly associated with poor sanitation due to poverty and poor infrastructure, increasing the risk of exposure to rodents. There are about three million rodents in NYC as of 2023, contributed by a high amount of garbage and dense population [16]. Ports of entry are mostly through abrasions or cuts of mucous membranes. Hence, high-risk occupations include sanitation workers, veterinarians, animal shelter workers, farm workers, hunters, laboratory technologists, and scientists handling animals. Although our patient did not recall the exact mechanism of his exposure, his occupation put him at high risk for contact with contaminated urine. Proper disposal of garbage plays a significant role in reducing rodent infestations. Long-term usage of rodenticides is not recommended as it is hazardous to children and wildlife, although it is helpful for temporary measures [3]. Following the strict protocol of occupational health and safety regulations, performing local risk assessments and training can reduce the risk of exposure. Appropriate protective equipment, such as gloves, boots, and goggles, for high-risk workers is crucial to prevent exposure of mucous membranes and skin to contaminants. Outbreaks also occur during the flooding, hurricanes, and monsoon season, where there is an abundance of rain [17]. Public education on disease symptoms, complications, and preventive measures including flood control projects is an essential component of active intervention efforts.

## Conclusions

This case emphasizes the importance of considering leptospirosis in the differential diagnosis of acute febrile illness with multiorgan involvement, even in non-endemic urban settings like New York, particularly in patients with potential occupational exposure to rodents. Weil’s disease can lead to rapid deterioration with renal, hepatic, and pulmonary complications. Our case also highlights that full renal recovery is possible despite delayed initiation and prolonged use of hemodialysis. Clinicians should maintain a high index of suspicion for leptospirosis, especially as climate change, urbanization, and extreme weather events may increase the risk of outbreaks even in temperate regions. Early recognition, appropriate antimicrobial therapy, and prompt supportive care, including dialysis and respiratory support, are key to a better prognosis and improved outcomes.

## References

[1] Costa F, Hagan JE, Calcagno J (2015). Global morbidity and mortality of leptospirosis: a systematic review. PLoS Negl Trop Dis.

[2] Torgerson PR, Hagan JE, Costa F (2015). Global burden of leptospirosis: estimated in terms of disability adjusted life years. PLoS Negl Trop Dis.

[3] Haake DA, Levett PN (2015). Leptospirosis in humans. Curr Top Microbiol Immunol.

[4] (2025). Leptospirosis: epidemiology, microbiology, clinical manifestations, and diagnosis. https://www.uptodate.com/contents/leptospirosis-epidemiology-microbiology-clinical-manifestations-and-diagnosis.

[5] (2024). 2024 health advisory # 10: continued increase in leptospirosis cases in New York City. https://www.nyc.gov/assets/doh/downloads/pdf/han/advisory/2024/han-advisory-10.pdf.

[6] Nakamura S (2022). Motility of the zoonotic spirochete Leptospira: insight into association with pathogenicity. Int J Mol Sci.

[7] Daher ED, de Abreu KL, da Silva Junior GB (2010). Leptospirosis-associated acute kidney injury. Braz J Nephrol.

[8] Araujo ER, Seguro AC, Spichler A, Magaldi AJ, Volpini RA, De Brito T (2010). Acute kidney injury in human leptospirosis: an immunohistochemical study with pathophysiological correlation. Virchows Arch.

[9] Cagliero J, Villanueva SY, Matsui M (2018). Leptospirosis pathophysiology: into the storm of cytokines. Front Cell Infect Microbiol.

[10] Pinto GV, Senthilkumar K, Rai P, Kabekkodu SP, Karunasagar I, Kumar BK (2022). Current methods for the diagnosis of leptospirosis: issues and challenges. J Microbiol Methods.

[11] Musso D, La Scola B (2013). Laboratory diagnosis of leptospirosis: a challenge. J Microbiol Immunol Infect.

[12] Samrot AV, Sean TC, Bhavya KS (2021). Leptospiral infection, pathogenesis and its diagnosis-a review. Pathogens.

[13] Andrade L, Cleto S, Seguro AC (2007). Door-to-dialysis time and daily hemodialysis in patients with leptospirosis: impact on mortality. Clin J Am Soc Nephrol.

[14] Julien M, Lombardi Y, Jabot J, Rafat C (2023). Impact of early renal replacement therapy in leptospirosis on mortality and long-term renal function: a retrospective analysis over 11 years in Reunion Island. FR-PO139. J Am Soc Nephrol.

[15] Chou LF, Yang HY, Hung CC, Tian YC, Hsu SH, Yang CW (2023). Leptospirosis kidney disease: evolution from acute to chronic kidney disease. Biomed J.

[16] (2025). There are 3 million rats in NYC, a 50% increase since 2010. https://mandmpestcontrol.com/pests/rats/3-million-rats-in-nyc/.

[17] Naing C, Reid SA, Aye SN, Htet NH, Ambu S (2019). Risk factors for human leptospirosis following flooding: a meta-analysis of observational studies. PLoS One.

